# Pattern of Response to Bronchial Challenge with Histamine in Patients with Non-Atopic Cough-Variant and Classic Asthma

**DOI:** 10.3390/jcm7070174

**Published:** 2018-07-12

**Authors:** Vladimir Zugic, Natasa Mujovic, Sanja Hromis, Jelena Jankovic, Mirjana Drvenica, Aleksandra Perovic, Ivan Kopitovic, Aleksandra Ilic, Dejan Nikolic

**Affiliations:** 1 Clinic for Pulmology, Clinical Center of Serbia, 11000 Belgrade, Serbia; vladimir.zugic@kcs.ac.rs (V.Z.); jelenajankovic@gmail.com (J.J.); sanjadudvarski@yahoo.com (A.I.); 2 Faculty of Medicine, University of Belgrade, 11000 Belgrade, Serbia; aleksandra.perovic90@gmail.com (A.P.); denikol27@gmail.com (D.N.); 3 Clinic for Physiotherapy and Rehabilitation, Clinical Center of Serbia, Pasterova 2, 11000 Belgrade, Serbia; 4 Institute for Pulmonary Diseases of Vojvodina, 21208 Sremska Kamenica, Serbia; shromis@gmail.com (S.H.); dmjovancevic@gmail.com (M.D.); ikopitovic@yahoo.com (I.K.); 5 Faculty of Medicine, University of Novi Sad, 21000 Novi Sad, Serbia; 6 Physical Medicine and Rehabilitation Department, University Children’s Hospital, 11000 Belgrade, Serbia

**Keywords:** non-atopic patients, cough-variant asthma, classic asthma, bronchoconstrictor, response pattern

## Abstract

Background: The aim of this study was to establish whether non-atopic patients with cough variant asthma (CVA) have different pattern of response to direct bronchoconstrictors than non-atopic patients with classic asthma (CA). Method: A total of 170 patients of both sexes with stable CVA and CA were screened for the study and 153 were included. Patients with proven atopy were not included and 17 patients with worsening of their condition or with verified bronchial obstruction during screening were excluded. All included patients performed spirometry and underwent a bronchial challenge with histamine according to long-standing protocol in our laboratory. Results: Significantly higher frequency of bronchial hyper-responsiveness (BHR) was found in patients with CA than in patients with CVA (63.9% vs. 44.9%, respectively; *p* < 0.05). Sensitivity was significantly lower in patients with CVA (*p* < 0.05), while no significant difference was found in maximal response and responsiveness. Only patients with positive challenge tests were included in the analysis. Conclusion: Adult non-atopic patients with CVA and CA have a pattern of response to non-specific bronchial stimuli similar to atopic patients with same conditions, with the exception of similar maximal response, which may reflect the efficacy of previous treatment. We believe that further studies are needed to clarify the mechanisms involved in airway response to non-specific stimuli in CVA and CA, especially in non-atopic patients. Further studies should also clarify whether this response pattern has any implications on clinical presentation or on treatment options.

## 1. Introduction

Cough variant asthma (CVA) was first described more than 40 years ago [1]. The only presenting symptom is isolated chronic cough, responsive to bronchodilator and/or inhaled corticosteroids therapy. The cough can occur for many years as an extremely annoying symptom interfering with work, sleep, and quality of life. Nearly 30% of cough variant asthma patients eventually develop wheezing, sometimes severe enough to require continuous treatment [2,3].

CVA is the only cause of chronic cough that is responsive to bronchodilators [4]. It is thus suggested that coughing as a result of CVA may be due to bronchoconstriction but the detailed causal relationship involved in cough and bronchoconstriction remains unknown. An animal experiment has suggested that cough due to bronchoconstriction is mediated via rapidly adapting receptors but not C fibers [5].

Compared to classic asthma, CVA shows similar levels of eosinophilic airway inflammation [6] and a milder degree of airway remodeling, such as sub-epithelial thickening, goblet cell hyperplasia, and vascular proliferation [7,8]. These changes may be secondary to airway inflammation but they may also be a consequence of long-term mechanical stimulation by coughing [4].

While the presence of bronchial hyper-responsiveness (BHR) is characteristic for CVA, it is merely consistent with it, but is not diagnostic [9]. Nevertheless, results of some studies indicate that an increase in BHR has a pathogenetic role in the development of wheezing during the course of CVA [6]. Patients with CVA showed milder BHR [10] when compared to patients with classic asthma (CA), and in both CA and atopic patients with CVA, BHR was inversely correlated with the percentage of sputum eosinophils [11].

However, most investigations that examined differences in response to bronchial stimuli between patients with CVA and CA included atopic patients, while few studies have been done investigating those differences in non-atopic patients with CVA and CA. Therefore, the aim of this study was to establish whether non-atopic patients with CVA have a different pattern of response to a direct bronchoconstrictor than the non-atopic patients with CA.

## 2. Material and Methods

### 2.1. Study Group

Non-atopic patients of both sexes with previously diagnosed CA and CVA from the Clinic for Pulmology, Clinical Center of Serbia, Belgrade, Serbia, and from the Institute for Pulmonary Disease of Vojvodina, Novi Sad, Serbia were included in our study. All patients signed informed consen, and the study was approved by Institution Review Boards of both institutions.

Inclusion criteria were:

- Age greater than 18 years
- Established diagnosis of CA or CVA in the previous 12 months
- Diagnosis of CA was made according to Global Initiative for Asthma (GINA) criteria [12]
- Diagnosis of CVA was based on: positive challenge testing, clinical improvement while on inhaled corticosteroid (ICS) treatment, and exclusion of other causes of chronic cough. All three criteria were fulfilled in patients with CVA.
- Stable diseases, without exacerbation in the 3 months prior to screening. GINA criteria for CA exacerbation were followed, while for CVA, exacerbation was defined by a board certified senior physician.
- Non-smoker or ex-smokers with smoking history of less than 10 pack/years
- Well-controlled chronicvasomotoric rhinitis
- Non-atopy, defined as negative prick skin testing in previous 6 months, normal values of serum immunoglobulin E (IgE), and normal blood eosinophils count.

Exclusion criteria were:

- Exacerbation of CA or CVA in the 3 months prior to screening
- Exacerbation of CA or CVA between screening and testing
- Exacerbation of chronic rhinitis before screening or between two visits
- Documented positive skin prick testing and/or eosinophylls (Eo) peripheral blood count greater than 150 cells/mm^3^, and or sIgE levels greater than 300 I/U
- Verified allergic rhinitis, seasonal or perennial
- Active smokers or ex-smokers with history of more than 10 pack/years
- Unwillingness to further participate in the study

On a screening visit, the study’s purpose and design were explained to the patients, who then signed informed consent. Patients without documented negative skin prick testing were not included. Clinical examination, spirometry, and blood sampling for Eo measurement were performed. Spirometry was performed in all patients on commercial Lilly pneumotachometer (MasterScreenPneumo, Viasys HealthCare, Höchberg, Germany) according to joint American Thoracic Society/European Respiratory Society standards [13]. Patients with bronchial obstruction defined as forced expiratory volume in first second (FEV1)/forced expiratory vital capacity (FVC) (FEV1/FVC) ratio less than 0.7 were excluded from the study and reversibility testing was performed.

Finally, a next visit was scheduled for seven days after (±2 days) and patients were instructed not to take medications that would influence bronchial challenge with histamine, such as antihistamines, anti-leukotriens, and theophylline and to abstain from short acting beta-2 agonist (SABA) or short acting muscarinic antagonist (SAMA) for 4 h before the visit and from long acting beta-2 agonist (LABA) or inhaled corticosteroids (ICS) = /LABA for 12 h before the visit. ICS and nasal corticosteroids (CS) were permitted in the period between two visits, as well as ICS/LABA combination in single inhaler up to 12 h before second visit.

On following visits, patients were checked for exacerbation of CA or CVA as well as chronic rhinitis between visits. Patients with exacerbation of either condition were excluded from the study. Next, blood Eo count was analyzed and patients with blood Eo count greater than 150 cells/mm^3^ were excluded from the study. The same was done for the serum immunoglobulin E (sIgE) and patients with sIgE levels greater than 300 IU were also exclude from the study. Next, spirometry was performed on the same spirometer as on the screening visit and according to same criteria. Patients with bronchial obstruction were excluded from the study and in these patients reversibility testing was performed. The remaining patients underwent bronchial challenge testing with histamine.

Bronchial challenge with histamine was performed on a commercial automatic provocation system (APS, Viasys HealthCare, Höchberg, Germany), which consisted of a breath-actuated nebulizer (Medicaid Pro, Baltimore, MA, USA) with a constant output of 480 mcg of histamine per nebulization, integrated with commercial Lilly pneumotachometer (MasterScreenPneumo, Viasys HealthCare, Höchberg, Germany) into a single computer-guided system. Previously, the nebulization sequence was programmed using original software, which, after nebulization of saline, gave an output of cumulative doses of histamine of 20, 60, 180, 580, and 2000 mcg. After every nebulization, spirometry was performed according to the previously mentioned standards.

Bronchial challenge was discontinued either after significant fall of FEV1 (20% or more) or when the challenge sequence was finished without significant response. At the end of the test each patient was given 4 puffs of combined ipratropium-bromide and fenoterol in single meter-dose inhaler (Berodual^®^ N, Boehringer Ingleheim Pharma GMBH & Co., Ingelheim, Germany) via spacer (Aerochamber^®^, Forest Laboratories Inc., New York, NY, USA), which gave a cumulative dose of 200 mcg and 80 mcg of fenoterol and ipratropium-bromide, respectively, whether significant fall of FEV1 was recorded or not.

Sensitivity was defined as cumulative dose in micrograms (mcg) of histamine that caused fall of FEV1 by 10% of pre-challenge value (PD10), and was calculated as follows:

where

D1—second to last dose of histamine before fall of FEV1 > 10%
D2—last dose of histamine after which fall of FEV1 > 10% was recorded
R1—Percentage of FEV1 fall after D1
R2—Percentage of FEV1 fall after D2

Responsiveness was defined as cumulative dose in mcg of histamine that caused fall of FEV1 by 20% of pre-challenge value (PD20) and was calculated as follows:

where

D1—second to last dose of histamine before fall of FEV1 > 20%
D2—last dose of histamine after which fall of FEV1 > 20% was recorded
R1—Percentage of FEV1 fall after D1
R2—Percentage of FEV1 fall after D2

Maximal response was defined as maximal fall of FEV1 during the challenge test expressed in percent.

### 2.2. Statistical Analysis

Continuous data were presented as mean values (MV) with standard deviation (SD), while categorical data were presented as whole numbers with percentages. For evaluation of statistical differences between observed groups of asthma patients, we performed a *t*-test for independent samples or a Mann-Whitney U test. Chi-square tests were used for statistical interpretation between different groups of asthma for categorical data. Differences were declared to be significant when *p* value was <0.05.

## 3. Results

In the period between January 2013 and January 2017, a total of 170 patients with CVA and CA were screened for the study and 153 were included, while 17 were excluded due to exacerbation between first and second visit. Demographics, history, and pre-challenge spirometry of included patients is presented in Table 1.

Four patients in the CA group had mild diabetes, while none did in the CVA group. Also, 5 patients in the CA group had well-controlled hypertension and only one patient did in the CVA group.

Significant differences were found in the male to female ratio, family history of asthma, Eo cell count, and presence of chronic rhinitis between the two groups of patients.

We did not find any difference in occurrence of CVA between sexes (Chi-square 2.215, *p* < 0.05).

**Figure 1:**
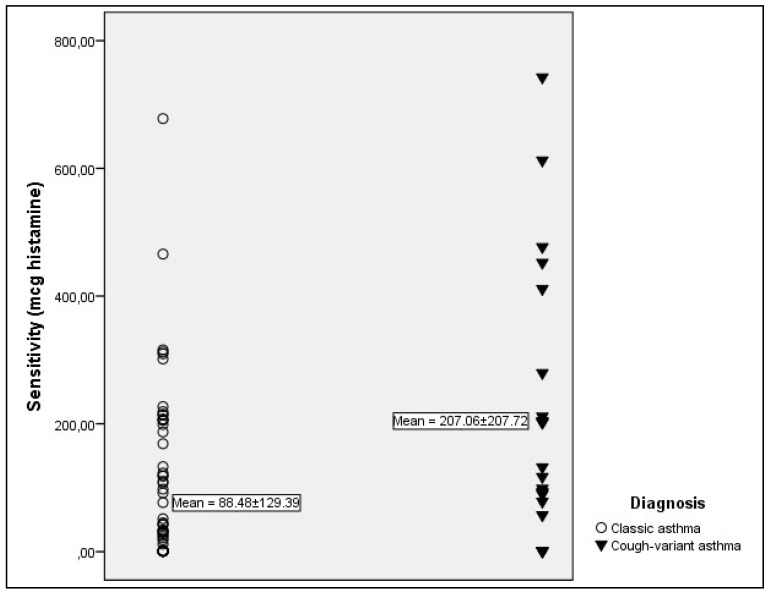
Sensitivity in classic asthma and cough-variant asthma in patients with cough-variant asthma (*p* < 0.05). Only patients with positive challenge testing were included.

**Figure 2:**
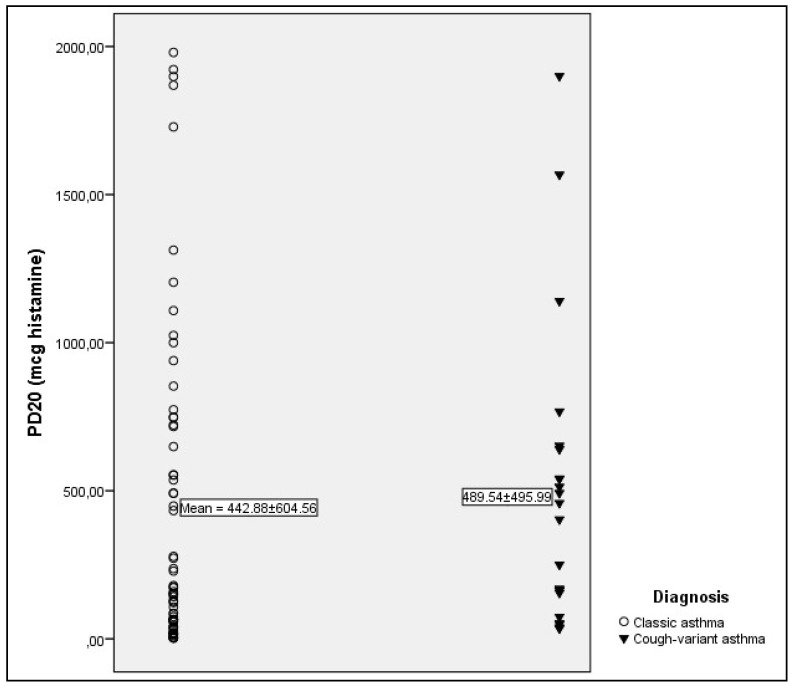
Responsiveness expressed as mean PD_20_ in (mcg) of histamine. No significant difference was found between groups (*p* > 0.05). Only patients with positive challenge testing were included.

**Figure 3:**
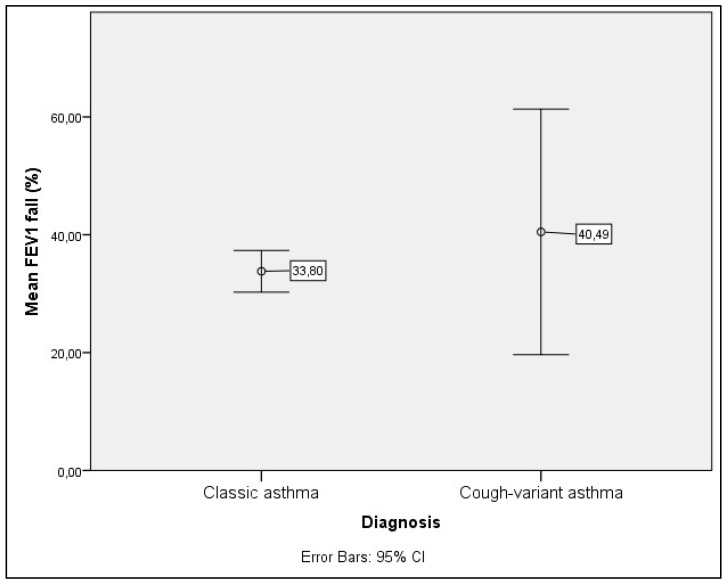
Maximal response to challenge presented as FEV1 fall in percent. No significant difference was found between groups (*p* > 0.05). Only patients with positive challenge testing were included.

Response to bronchial challenge in patients with CVA and CA is presented in Table 2 and Figure 1, Figure 2 and Figure 3.

Significantly higher frequency of BHR was found in patients with CA than in patients with CVA (63.9% vs. 44.9%, respectively; *p* < 0.05). Sensitivity was significantly lower in patients with CVA (*p* < 0.05) (Figure 1), while no significant difference was found in responsiveness (Figure 2) and maximal response (Figure 3).

We did not find any significant differences in PD10, PD20, and FEV1 fall in regard to sex for the presence of chronic vasomotoric rhinitis and family history of asthma (Supplemental Material Table S1).

## 4. Discussion

To our knowledge, this is among the first studies that addressed pattern of bronchial response to non-specific stimuli in non-atopic patients with CVA and CA. In our study it was significant that lower sensitivity during histamine challenge testing was found in patients with CVA compared to patients with CA, while we did not find any significant difference in frequencies of positive challenge tests, maximal response, and bronchial responsiveness.

Airway sensitivity is most likely associated with airway inflammation, epithelial damage or malfunction, abnormal neural control, and increased inflammatory cell number and activity. It is defined as position of the dose response curve during bronchial challenge [14,15]. As there isn’t any clearly established method for determining airway sensitivity, we defined sensitivity as a cumulative dose of histamine that caused a fall of FEV1 by 10% of the pre-challenge value. Previous studies defined sensitivity as a cumulative dose of methacholine at the inflection point of the respiratory resistance tracing [16]. This method was proposed more than 50 years ago [17] and has been used ever since. Since normal diurnal variation of FEV1 in persons without asthma is up to 10%, it is acknowledged that larger variation is of significance in establishing diagnoses of asthma [12]. Despite the difference in measuring response to challenge, both methods determine, in practical terms, the same thing: the point where response starts to be significant.

Previous studies have given conflicting results when sensitivity was compared between patients with CVA and CA. While in one, as in the present study, airway sensitivity was lower in patients with CVA than in those with classic asthma [8], in another no significant difference in airway sensitivity was found between these two conditions [18]. One possible explanation is the different types of patients selected for the studies. It seems that children with CVA or CA have similar airway sensitivity, which is not true for adult patients with these conditions, most likely because of difference in airway physiology in children and adults.

The maximal airway response on the dose–response curve to bronchoconstrictor stimuli seems to reflect the potential degree of airway obstruction in individual patients, irrespective of the level of sensitivity [19]. In children with CVA, future development of wheezing was in correlation with increased maximal response to bronchoconstrictor [20]. Koh et al. [6] suggested that it is the level of maximal airway response, rather than the degree of bronchial sensitivity, that is an important risk factor for the future development of classic asthma in patients with CVA. In our study, both groups of patients showed similar maximal response to bronchoconstrictor challenge, which probably reflects good control of both entities due to regular treatment. A previous study found significantly lower maximal airway response in CVA than in CA patients [18]. A possible reason for this discrepancy could be different pathophysiological mechanisms in CVA and CA.

A significantly higher percentage of patients with CA in our study had BHR but in those who had BHR, a similar level of responsiveness, defined as PD20, was found in both groups. It is possible that non-atopic patients with CVA have better response to anti-inflammatory treatment than patients with CA, or that different treatment options in our patients had different effects on BHR. Since we did not stratify our patients according to treatment´, we can only hypothesize.

In patients with CVA, there was a lack of association between cough sensitivity and bronchoconstrictor responsiveness, which implies different mechanisms that mediate cough production and modulate airway muscular tone [21]. Since we were interested only in BHR, we did not measure cough sensitivity during bronchial challenge. Therefore, we cannot draw any conclusion on a possible relationship between cough sensitivity and BHR in non-atopic patients with CVA. This was also the case for airway reactivity in our patients, since we did not evaluate the slope of the dose-response curve during bronchial challenge. A previous study showed that adults with CVA were significantly less reactive to methacholine than were those with classic asthma, although the difference in airway reactivity between the two groups was small [10]. This is also the case in children with CVA, who showed significantly decreased bronchial reactivity compared with the control subjects and children with classic asthma, which means that children with CVA demonstrated slower bronchoconstriction against nonspecific airway stimuli than the children with classic asthma [14]. Whether this is true for adults and children with non-atopic CVA and CA is not known.

We also did not take into account the possible influence of concomitant chronic rhinitis on pattern of response to bronchial challenge. However, since concomitant seasonal allergic rhinitis is not associated with disease severity and FEV1 in patients with classic asthma and CVA [22], it is possible that influence of chronic rhinitis on BHR in non-atopic patients with these conditions is not significant.

Finally, we have used only histamine for bronchial challenge in our laboratories for more than thirty years. Since histamine and methacholine, despite slight differences observed, are similar in action [15,23], we believe that our choice of challenge agent has’t influenced the results of this investigation.

## 5. Conclusions

Despite all aforementioned limitations of our study, we have shown that adult non-atopic patients with CVA and CA have a pattern of response to non-specific bronchial stimuli similar to atopic patients with same conditions, with the exception of similar maximal response, which likely reflects efficacy of previous treatment. While it is known that different inflammatory subtypes (eosinophyllic, neutrophyllic, mixed granulocytic and paucigranulocytic) in CVA show differences in eosinophil cationic protein (ECP) and interleukin 8 (IL-8) levels and also in maintenance doses of ICS, no such difference was found in airway responsiveness [24]. Therefore, there is still a question as to which characteristic of non-atopy is responsible for the differences in response to bronchial challenge found in our and previous studies, since it is obvious that the type of inflammatory cells is not.

Given the facts above, we believe that further studies are needed to clarify mechanisms involved in airway response to non-specific stimuli in CVA and CA, especially in non-atopic patients. Further studies should also clarify whether this response pattern has any implications on clinical presentation or on treatment options.

## Figures and Tables

**Table 1:** Demographics, medical history, eosinophylls (Eo), serum immunoglobulin E (sIgE), BMI, mean daily dose of inhaled corticosteroids (ICS) and pre-challenge spirometry of included patients.

Parameter	Cough-Variant Asthma (*n* = 49)	“Classic” Asthma (*n* = 108)	*p*
Age (years, MV ± SD)	40.84 ± 11.39	37.52 ± 11.24	>0.05 ^a^
Age of asthma diagnosis (years, MV ± SD)	30.53 ± 10.34	28.17 ± 9.51	>0.05 ^a^
Males (*n*, percent)	11, 22.4%	37, 34.3%	<0.01 ^b^
Females (*n*, percent)	38, 77.6%	71, 65.7%	<0.01 ^b^
Family history of asthma (*n*, percent)	10, 20.4%	29, 26.9%	<0.01 ^b^
Chronic vasomotoric rhinitis (*n*, percent)	18, 36.7%	61, 56.5%	<0.01 ^b^
Eo (cells/mm^3^, MV ± SD)	116.69 ± 27.25	103.10 ± 27.68	<0.05 ^a^
sIgE (IU, MV ± SD)	112.18 ± 35.43	111.99 ± 36.15	>0.05 ^a^
BMI (kg/m^2^, MV ± SD)	26.11 ± 4.69	24.73 ± 4.09	>0.05 ^a^
Daily ICS dose (mcg budesonide equivalent, MV ± SD)	289.79 ± 100.5	283.33 ± 99.06	>0.05 ^a^
FVC (% of predicted, MV ± SD)	101.04 ± 6.33	102.04 ± 6.34	>0.05 ^a^
FEV_1_ (% o fpredicted, MV ± SD)	94.57 ± 5.81	95.97 ± 5.93	>0.05 ^a^

^a^*t*-test for independent samples; ^b^ Chi square.

**Table 2:** Bronchial challenge in included patients.

Parameter	Cough-Variant Asthma (*N* = 49)	“Classic” Asthma (*N* = 108)	*p*
Positive challenge (*n*, percent)	22, 44.9%	69, 63.9%	<0.05 ^a^
PD10 (mcg, MV ± SD)	207.06 ± 207.72	88.48 ± 129.39	<0.05 ^b^
PD20 (mcg, MV ± SD)	489.54 ± 495.99	442.88 ± 604.56	>0.05 ^b^
FEV_1_ fall (percent, MV ± SD)	40.48 ± 46.98	33.80 ± 14.75	>0.05 ^b^

^a^ Mann-Whitney U test; ^b^ *t*-test for independent samples; only patients with positive challenge testing were included.

## Supplementary Materials

Supplementary Material is available online at http://www.mdpi.com/2077-0383/7/7/174/s1.
