# Supplementary material for: Pattern of Response to Bronchial Challenge with Histamine in Patients with Non-Atopic Cough-Variant and Classic Asthma
Source: J Clin Med. 2018 Jul 12;7(7):174. doi: 10.3390/jcm7070174 (PMC6069128; doi:10.3390/jcm7070174)
Supplement: Supplementary file 1 [file jcm-07-00174-s001.pdf]

Table 1. Differencesof PD10, PD20 andpercentageof FEV1 fall in regard to sexpresenceofchronicvasomotoricrhinitisandfamilyhistoryofasthma

| | PD10 | | PD20 | | $FEV_1$fall | |
|---|---|---|---|---|---|---|
| | mcg, MV±SD | p* | mcg, MV±SD | p* | percent, MV±SD | p* |
| Sex | | | | | | |
| Male | 102.40±168.96 | >0.05 | 409.25±603.30 | >0.05 | 30.44±11.70 | >0.05 |
| Female | 124.71±156.99 | | 440.97±501.40 | | 37.92±30.61 | |
| Chronicvasomotoricrhinitis | | | | | | |
| Yes | | | | | | |
| No | 126.46±164.55 | >0.05 | 451.17±535.23 | >0.05 | 30.93±22.69 | >0.05 |
| | 106.63±154.93 | | 404.62±529.23 | | 28.53±11.62 | |
| Familyhistoryofasthma | | | | | | |
| Yes | 147.50±181.63 | >0.05 | 578.70±645.70 | >0.05 | 33.97±14.24 | >0.05 |
| No | 110.57±154.45 | | 393.78±494.55 | | 36.16±29.01 | |

* Independentsample t-test. Onlypatientswithpositive BCT wereincluded
